# (1,4,7,10,13,16-Hexaoxacyclo­octa­deca­ne)dimethyl­indium(III) trifluoro­methane­sulfonate

**DOI:** 10.1107/S1600536811001899

**Published:** 2011-01-22

**Authors:** Benjamin F. T. Cooper, Charles L. B. Macdonald

**Affiliations:** a Department of Chemistry and Biochemistry, University of Windsor, Windsor, Ontario, Canada N9B 3P4

## Abstract

In the title compound, [In(CH_3_)_2_(C_12_H_24_O_6_)](CF_3_O_3_S), two of the In—O distances within the cation are significantly shorter than the other four. The In^III^ atom is in a distorted hexa­gonal–bipyramidal coordination geometry in which the C—In—C angle is 175.44 (12)°. The crystal structure is stabilized by weak inter­molecular C—H⋯O hydrogen bonds.

## Related literature

For the preparation of [In][OTf], where OTf = trifluoro­methane­sulfonate, see: Macdonald *et al.* (2004 ▶); Cooper & Macdonald (2010 ▶). For the preparation of the crowned complex [In([18]crown-6][OTf], see: Andrews & Macdonald (2005 ▶). For the oxidative addition of [In([18]crown-6][OTf] into aliphatic C—Cl bonds, see: Cooper *et al.* (2007 ▶). For the reaction of [In][OTf] with indium trihalides, see: Cooper *et al.* (2011 ▶). For the structure of the related ‘base-free’ salt [InMe_2_][Br], see: Hausen *et al.* (1975 ▶). For the structure of a related (β-diketonate)InMe_2_ complex, see: Xu *et al.* (2000 ▶). For a description of the Cambridge Structural Database, see: Allen (2002 ▶).
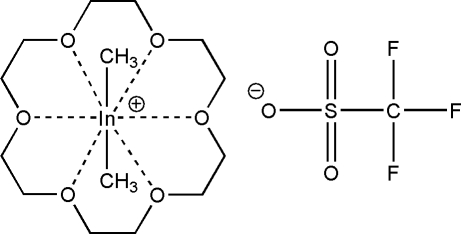


## Experimental

### 

#### Crystal data

[In(CH_3_)_2_(C_12_H_24_O_6_)](CF_3_O_3_S)
*M* *_r_* = 558.27
Monoclinic, 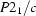

*a* = 12.9580 (19) Å
*b* = 12.7242 (19) Å
*c* = 14.683 (2) Å
β = 112.801 (2)°
*V* = 2231.8 (6) Å^3^
*Z* = 4
Mo *K*α radiation
μ = 1.22 mm^−1^
*T* = 173 K
0.20 × 0.10 × 0.10 mm

#### Data collection

Bruker APEX diffractometer
Absorption correction: multi-scan (*SADABS*; Bruker, 2000 ▶) *T*_min_ = 0.793, *T*_max_ = 0.888
24322 measured reflections
5073 independent reflections
3744 reflections with *I* > 2σ(*I*)
*R*_int_ = 0.050

#### Refinement

*R*[*F* ^2^ > 2σ(*F* ^2^)] = 0.037
*wR*(*F* ^2^) = 0.077
*S* = 1.02
5073 reflections
264 parameters
H-atom parameters constrained
Δρ_max_ = 0.72 e Å^−3^
Δρ_min_ = −0.34 e Å^−3^

### 

Data collection: *SMART* (Bruker, 1997 ▶); cell refinement: *SAINT* (Bruker, 1997 ▶); data reduction: *SAINT*; program(s) used to solve structure: *SIR97* (Altomare *et al.*, 1999 ▶); program(s) used to refine structure: *SHELXL97* (Sheldrick, 2008 ▶); molecular graphics: *SHELXTL* (Sheldrick, 2008 ▶); software used to prepare material for publication: *SHELXTL*, *PLATON* (Spek, 2009 ▶) and *WinGX* (Farrugia, 1999 ▶).

## Supplementary Material

Crystal structure: contains datablocks global, I. DOI: 10.1107/S1600536811001899/lh5188sup1.cif

Structure factors: contains datablocks I. DOI: 10.1107/S1600536811001899/lh5188Isup2.hkl

Additional supplementary materials:  crystallographic information; 3D view; checkCIF report

## Figures and Tables

**Table 1:** Selected bond lengths (Å)

In—C2	2.094 (3)
In—C1	2.098 (3)
In—O1	2.7145 (19)
In—O2	2.620 (2)
In—O3	2.660 (2)
In—O4	2.816 (2)
In—O5	2.7810 (19)
In—O6	2.842 (2)

**Table 2:** Hydrogen-bond geometry (Å, °)

*D*—H⋯*A*	*D*—H	H⋯*A*	*D*⋯*A*	*D*—H⋯*A*
C13—H13*B*⋯O22^i^	0.99	2.54	3.455 (4)	153
C14—H14*A*⋯O23^ii^	0.99	2.43	3.348 (4)	155
C17—H17*A*⋯O5^iii^	0.99	2.58	3.527 (4)	160
C19—H19*A*⋯O23^iv^	0.99	2.53	3.322 (4)	137

Symmetry codes: (i) 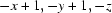; (ii) 
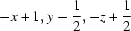
; (iii) 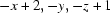; (iv) 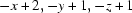.

**Fig. 1.**
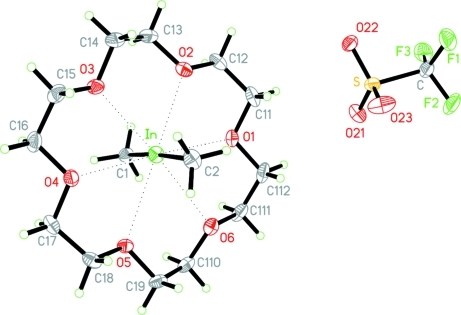
Thermal ellipsoid plot (30% probability surface) of the contents of the asymmetric unit of [InMe2([18]crown-6)][O3SCF3] (1).

**Fig. 2.**
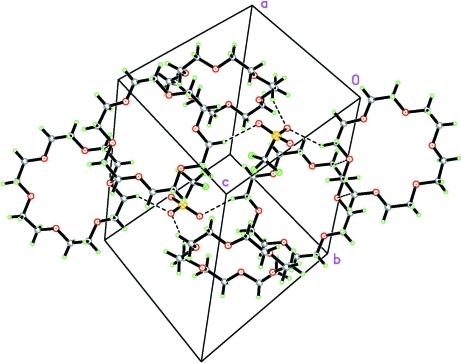
Partial packing diagram of [InMe2([18]crown-6)][O3SCF3] (1) illustrating the intramolecular H-bonding interactions. The InMe2 fragments are removed for clarity.

## supplementary crystallographic information

### Comment

In contrast to the mixed-valent compounds obtained through the reaction of the crown ether adduct of indium(I) trifluoromethanesulfonate, [In([18]crown-6)][OTf], with indium trihalides (Cooper *et al.*, in press), the treatment of [In([18]crown-6)][OTf] with trimethylindium results in the formation of the organometallic salt [InMe_2_([18]crown-6)][OTf] (1), as the only isolable crystalline product.

The asymmetric unit of the title compound (1) is shown in Fig. 1. The In–C distances in the cation in 1 of 2.094 (3) and 2.098 (3) Å are somewhat shorter thant the mean distance of 2.151 Å for InMe_2_ fragments coordinated by at least two oxygen atoms that have been reported in the Cambridge Structural Database (as determined from the total of 38 examples that are found in CSD Version 5.31) (Allen, 2002). Furthermore, the nearly linear C–In–C fragment in the cation, although anticipated because of the geometry of the crown ether ligand and observed in "base-free" salt [InMe_2_][Br] (Hausen *et al.*, 1975), is a very unusual arrangement for coordinated InMe_2_ moieties: the largest angle reported in the CSD sample cited above is 159.6 (2)° for a dimeric structure featuring a β-diketonate ligand (Xu *et al.*, 2000). Overall, the coordination geometry of the indium center in 1 is best-described as being a slightly-distorted hexagonal bipyramid in which the metal atom is appreciably closer to two of the oxygen atoms in the ligand. As such, the geometry is reminscent of those exhibited by the products derived from the oxidative addition of chloro-alkanes to benzannelated derivatives of [In([18]crown-6)][OTf] (Cooper *et al.*, 2007).

The indistinguishable S–O distances within each of the trifluoromethanesulfonate anions are consistent with the completely delocalized structure expected for an unperturbed "ionic" trifluoromethanesulfonate anion. In the crystal structure there are weak C—H···O≐S hydrogen bonds (see Table 2) which link the anions and the cations. In addition, there are further weak intermolecular C—H···O hydrogen bonds between symmetry related crown ether ligands (Fig. 2)

### Experimental

The salt [InMe_2_(18-crown-6)][OTf] was obtained in high yield from the reaction of trimethylindium with the crown ether adduct of indium(I) trifluoromethanesulfonate that was prepared as described previously (Andrews & Macdonald, 2005; Cooper & Macdonald, 2010). Suitable crystals were obtained by the slow evaporation of a dichloromethane solution of the salt in a nitrogen-filled glove box.

### Refinement

H atoms were initially located in difference Fourier maps but were subsequently modeled as riding atoms with a C–H distance of 0.98Å and U_iso_(H) of 1.5 times the U_eq_(C) of the carbon atom to which they are attached for each methyl hydrogen atom and with a C–H distance of 0.99Å and U_iso_(H) of 1.2 times the U_eq_(C) of the carbon atom to which they are attached for each methylene hydrogen atom.

### Crystal data

**Table:**

[In(CH_3_)_2_(C_12_H_24_O_6_)](CF_3_O_3_S)	*F*(000) = 1136
*M**_r_* = 558.27	*D*_x_ = 1.662 Mg m^−^^3^
Monoclinic, *P*2_1_/*c*	Melting point: 428 K
Hall symbol: -P 2ybc	Mo *K*α radiation, λ = 0.71073 Å
*a* = 12.9580 (19) Å	Cell parameters from 7679 reflections
*b* = 12.7242 (19) Å	θ = 2.2–28.0°
*c* = 14.683 (2) Å	µ = 1.22 mm^−^^1^
β = 112.801 (2)°	*T* = 173 K
*V* = 2231.8 (6) Å^3^	Prism, colourless
*Z* = 4	0.20 × 0.10 × 0.10 mm

### Data collection

**Table:**

Bruker APEX diffractometer	5073 independent reflections
Radiation source: fine-focus sealed tube	3744 reflections with *I* > 2σ(*I*)
graphite	*R*_int_ = 0.050
φ and ω scans	θ_max_ = 27.5°, θ_min_ = 1.7°
Absorption correction: multi-scan (*SADABS*; Bruker, 2000)	*h* = −16→16
*T*_min_ = 0.793, *T*_max_ = 0.888	*k* = −16→16
24322 measured reflections	*l* = −19→19

### Refinement

**Table:**

Refinement on *F*^2^	Primary atom site location: structure-invariant direct methods
Least-squares matrix: full	Secondary atom site location: difference Fourier map
*R*[*F*^2^ > 2σ(*F*^2^)] = 0.037	Hydrogen site location: inferred from neighbouring sites
*wR*(*F*^2^) = 0.077	H-atom parameters constrained
*S* = 1.02	*w* = 1/[σ^2^(*F*_o_^2^) + (0.0344*P*)^2^ + 0.2258*P*] where *P* = (*F*_o_^2^ + 2*F*_c_^2^)/3
5073 reflections	(Δ/σ)_max_ = 0.001
264 parameters	Δρ_max_ = 0.72 e Å^−^^3^
0 restraints	Δρ_min_ = −0.34 e Å^−^^3^

### Special details

**Table:**

Experimental. The data crystal was coated in mineral oil and placed rapidly in the cold nitrogen stream of the Kryoflex low-temperature device.Spectroscopic and physical data: dp 155 °C ^1^H NMR (C_6_D_6_): δ= 3.057 (s; CH_2_, 24H), δ= -0.021 (s, CH_3_, 6H) ^13^C NMR (C_6_D_6_): δ= 70.00 (s; CH_2_), -2.491 (s, CH_3_) ^19^F NMR (C_6_D_6_):δ= 78.12
Geometry. All e.s.d.'s (except the e.s.d. in the dihedral angle between two l.s. planes) are estimated using the full covariance matrix. The cell e.s.d.'s are taken into account individually in the estimation of e.s.d.'s in distances, angles and torsion angles; correlations between e.s.d.'s in cell parameters are only used when they are defined by crystal symmetry. An approximate (isotropic) treatment of cell e.s.d.'s is used for estimating e.s.d.'s involving l.s. planes.
Refinement. Refinement of *F*^2^ against ALL reflections. The weighted *R*-factor *wR* and goodness of fit *S* are based on *F*^2^, conventional *R*-factors *R* are based on *F*, with *F* set to zero for negative *F*^2^. The threshold expression of *F*^2^ > σ(*F*^2^) is used only for calculating *R*-factors(gt) *etc*. and is not relevant to the choice of reflections for refinement. *R*-factors based on *F*^2^ are statistically about twice as large as those based on *F*, and *R*- factors based on ALL data will be even larger.

### Fractional atomic coordinates and isotropic or equivalent isotropic displacement parameters (Å^2^)

**Table:**

	*x*	*y*	*z*	*U*_iso_*/*U*_eq_	
In	0.788938 (19)	0.230045 (18)	0.358803 (16)	0.03526 (8)	
C1	0.8733 (2)	0.1496 (2)	0.2835 (2)	0.0330 (7)	
H1A	0.9488	0.1787	0.3024	0.050*	
H1B	0.8321	0.1576	0.2122	0.050*	
H1C	0.8784	0.0749	0.3009	0.050*	
C2	0.7168 (3)	0.3162 (3)	0.4405 (2)	0.0431 (8)	
H2A	0.7662	0.3142	0.5106	0.065*	
H2B	0.6441	0.2856	0.4315	0.065*	
H2C	0.7064	0.3893	0.4176	0.065*	
C11	0.7141 (3)	0.4506 (2)	0.2018 (2)	0.0456 (9)	
H11A	0.7277	0.5074	0.1617	0.055*	
H11B	0.6758	0.4811	0.2423	0.055*	
C12	0.6440 (3)	0.3674 (3)	0.1367 (2)	0.0422 (8)	
H12A	0.5725	0.3975	0.0903	0.051*	
H12B	0.6834	0.3353	0.0977	0.051*	
C13	0.5558 (3)	0.2066 (3)	0.1395 (2)	0.0492 (9)	
H13A	0.5993	0.1649	0.1097	0.059*	
H13B	0.4892	0.2356	0.0855	0.059*	
C14	0.5210 (3)	0.1392 (3)	0.2044 (3)	0.0485 (9)	
H14A	0.4782	0.1811	0.2347	0.058*	
H14B	0.4725	0.0816	0.1656	0.058*	
C15	0.5917 (3)	0.0237 (3)	0.3400 (2)	0.0455 (8)	
H15A	0.5402	−0.0306	0.2983	0.055*	
H15B	0.5540	0.0603	0.3781	0.055*	
C16	0.6968 (3)	−0.0260 (3)	0.4084 (2)	0.0464 (9)	
H16A	0.6801	−0.0780	0.4510	0.056*	
H16B	0.7345	−0.0628	0.3704	0.056*	
C17	0.8681 (3)	0.0124 (2)	0.5378 (2)	0.0396 (8)	
H17A	0.9091	−0.0263	0.5038	0.048*	
H17B	0.8511	−0.0371	0.5821	0.048*	
C18	0.9371 (3)	0.1009 (2)	0.5957 (2)	0.0395 (8)	
H18A	0.8946	0.1419	0.6269	0.047*	
H18B	1.0061	0.0738	0.6485	0.047*	
C19	1.0489 (3)	0.2400 (2)	0.5817 (2)	0.0397 (8)	
H19A	1.1161	0.2036	0.6284	0.048*	
H19B	1.0207	0.2883	0.6197	0.048*	
C110	1.0775 (3)	0.3001 (3)	0.5072 (2)	0.0427 (8)	
H11C	1.1408	0.3484	0.5410	0.051*	
H11D	1.0999	0.2512	0.4657	0.051*	
C111	1.0032 (3)	0.4257 (3)	0.3795 (2)	0.0477 (9)	
H11E	1.0296	0.3843	0.3357	0.057*	
H11F	1.0616	0.4777	0.4155	0.057*	
C112	0.8974 (3)	0.4799 (2)	0.3207 (2)	0.0490 (9)	
H11G	0.8686	0.5175	0.3651	0.059*	
H11H	0.9104	0.5320	0.2762	0.059*	
O1	0.81800 (17)	0.40389 (15)	0.26423 (15)	0.0354 (5)	
O2	0.62296 (17)	0.28979 (16)	0.19748 (15)	0.0373 (5)	
O3	0.61913 (16)	0.09713 (16)	0.27933 (15)	0.0363 (5)	
O4	0.76689 (16)	0.05488 (15)	0.46713 (14)	0.0347 (5)	
O5	0.96498 (16)	0.16552 (15)	0.52962 (13)	0.0323 (5)	
O6	0.98188 (17)	0.35800 (16)	0.44774 (15)	0.0387 (5)	
O21	0.80907 (19)	0.71444 (18)	0.16120 (17)	0.0495 (6)	
O22	0.6098 (2)	0.68156 (18)	0.08395 (16)	0.0535 (6)	
O23	0.6906 (2)	0.7465 (2)	0.25086 (18)	0.0654 (8)	
F1	0.57677 (19)	0.90759 (17)	0.1002 (2)	0.0820 (8)	
F2	0.75310 (19)	0.93621 (17)	0.17324 (19)	0.0772 (7)	
F3	0.68476 (18)	0.88076 (16)	0.02420 (17)	0.0666 (6)	
S	0.69897 (7)	0.73809 (6)	0.15687 (5)	0.03685 (19)	
C	0.6768 (3)	0.8726 (3)	0.1110 (3)	0.0494 (9)	

### Atomic displacement parameters (Å^2^)

**Table:**

	*U*^11^	*U*^22^	*U*^33^	*U*^12^	*U*^13^	*U*^23^
In	0.03894 (14)	0.03720 (13)	0.03436 (13)	0.00538 (11)	0.01936 (10)	−0.00321 (10)
C1	0.0324 (17)	0.0337 (17)	0.0331 (16)	0.0000 (13)	0.0129 (14)	−0.0078 (13)
C2	0.053 (2)	0.0439 (19)	0.0409 (18)	0.0110 (17)	0.0272 (17)	0.0003 (15)
C11	0.060 (2)	0.0338 (19)	0.052 (2)	0.0159 (17)	0.0313 (19)	0.0140 (16)
C12	0.043 (2)	0.048 (2)	0.0364 (18)	0.0160 (16)	0.0161 (16)	0.0163 (15)
C13	0.040 (2)	0.056 (2)	0.0371 (18)	−0.0015 (17)	−0.0018 (15)	0.0043 (16)
C14	0.0288 (18)	0.054 (2)	0.053 (2)	−0.0025 (16)	0.0057 (16)	0.0021 (17)
C15	0.044 (2)	0.048 (2)	0.047 (2)	−0.0175 (17)	0.0200 (17)	−0.0018 (16)
C16	0.064 (2)	0.0342 (19)	0.044 (2)	−0.0112 (17)	0.0240 (18)	0.0032 (15)
C17	0.0420 (19)	0.0366 (18)	0.0448 (19)	0.0124 (15)	0.0219 (16)	0.0134 (15)
C18	0.0403 (19)	0.050 (2)	0.0293 (17)	0.0094 (15)	0.0143 (15)	0.0098 (14)
C19	0.0355 (17)	0.049 (2)	0.0287 (16)	−0.0009 (15)	0.0060 (14)	−0.0126 (14)
C110	0.0354 (18)	0.046 (2)	0.0451 (19)	−0.0086 (15)	0.0136 (16)	−0.0186 (15)
C111	0.060 (2)	0.044 (2)	0.043 (2)	−0.0247 (18)	0.0241 (18)	−0.0130 (16)
C112	0.079 (3)	0.0262 (18)	0.048 (2)	−0.0132 (18)	0.031 (2)	−0.0051 (15)
O1	0.0453 (13)	0.0246 (11)	0.0404 (12)	0.0016 (9)	0.0211 (11)	0.0002 (9)
O2	0.0350 (12)	0.0428 (14)	0.0315 (11)	0.0043 (10)	0.0101 (9)	0.0071 (9)
O3	0.0275 (11)	0.0436 (13)	0.0363 (12)	−0.0050 (9)	0.0108 (9)	0.0028 (10)
O4	0.0363 (12)	0.0298 (12)	0.0376 (12)	0.0005 (9)	0.0140 (10)	0.0028 (9)
O5	0.0344 (11)	0.0375 (12)	0.0233 (10)	−0.0001 (9)	0.0093 (9)	−0.0013 (9)
O6	0.0425 (13)	0.0362 (12)	0.0413 (13)	−0.0055 (10)	0.0204 (11)	−0.0040 (10)
O21	0.0496 (15)	0.0500 (15)	0.0544 (15)	0.0134 (12)	0.0260 (12)	0.0138 (12)
O22	0.0612 (16)	0.0355 (13)	0.0443 (14)	−0.0113 (12)	−0.0010 (12)	0.0012 (11)
O23	0.0481 (15)	0.113 (2)	0.0387 (14)	−0.0225 (14)	0.0207 (12)	−0.0071 (14)
F1	0.0524 (14)	0.0537 (14)	0.133 (2)	0.0146 (11)	0.0279 (15)	−0.0114 (14)
F2	0.0667 (15)	0.0492 (14)	0.1039 (19)	−0.0221 (12)	0.0202 (14)	−0.0338 (13)
F3	0.0696 (15)	0.0532 (14)	0.0690 (15)	−0.0031 (11)	0.0181 (12)	0.0226 (11)
S	0.0349 (4)	0.0409 (5)	0.0307 (4)	−0.0038 (3)	0.0082 (3)	0.0001 (3)
C	0.041 (2)	0.037 (2)	0.065 (2)	−0.0057 (16)	0.0147 (18)	−0.0129 (18)

### Geometric parameters (Å, °)

**Table:**

In—C2	2.094 (3)	C16—O4	1.422 (4)
In—C1	2.098 (3)	C16—H16A	0.9900
In—O1	2.7145 (19)	C16—H16B	0.9900
In—O2	2.620 (2)	C17—O4	1.426 (3)
In—O3	2.660 (2)	C17—C18	1.485 (4)
In—O4	2.816 (2)	C17—H17A	0.9900
In—O5	2.7810 (19)	C17—H17B	0.9900
In—O6	2.842 (2)	C18—O5	1.421 (3)
C1—H1A	0.9800	C18—H18A	0.9900
C1—H1B	0.9800	C18—H18B	0.9900
C1—H1C	0.9800	C19—O5	1.421 (3)
C2—H2A	0.9800	C19—C110	1.495 (4)
C2—H2B	0.9800	C19—H19A	0.9900
C2—H2C	0.9800	C19—H19B	0.9900
C11—O1	1.431 (4)	C110—O6	1.415 (4)
C11—C12	1.479 (5)	C110—H11C	0.9900
C11—H11A	0.9900	C110—H11D	0.9900
C11—H11B	0.9900	C111—O6	1.427 (4)
C12—O2	1.426 (3)	C111—C112	1.478 (5)
C12—H12A	0.9900	C111—H11E	0.9900
C12—H12B	0.9900	C111—H11F	0.9900
C13—O2	1.424 (4)	C112—O1	1.421 (4)
C13—C14	1.477 (5)	C112—H11G	0.9900
C13—H13A	0.9900	C112—H11H	0.9900
C13—H13B	0.9900	O21—S	1.435 (2)
C14—O3	1.424 (4)	O22—S	1.428 (2)
C14—H14A	0.9900	O23—S	1.429 (2)
C14—H14B	0.9900	F1—C	1.320 (4)
C15—O3	1.427 (3)	F2—C	1.329 (4)
C15—C16	1.485 (4)	F3—C	1.322 (4)
C15—H15A	0.9900	S—C	1.821 (4)
C15—H15B	0.9900		
			
C2—In—C1	175.44 (12)	O4—C16—H16A	110.2
C2—In—O2	88.49 (10)	C15—C16—H16A	110.2
C1—In—O2	94.48 (9)	O4—C16—H16B	110.2
C2—In—O3	96.16 (11)	C15—C16—H16B	110.2
C1—In—O3	88.28 (9)	H16A—C16—H16B	108.5
O2—In—O3	62.50 (6)	O4—C17—C18	108.0 (2)
C2—In—O1	92.72 (10)	O4—C17—H17A	110.1
C1—In—O1	85.66 (9)	C18—C17—H17A	110.1
O2—In—O1	61.91 (6)	O4—C17—H17B	110.1
O3—In—O1	123.29 (6)	C18—C17—H17B	110.1
C2—In—O5	91.73 (10)	H17A—C17—H17B	108.4
C1—In—O5	85.31 (9)	O5—C18—C17	107.7 (2)
O2—In—O5	179.69 (6)	O5—C18—H18A	110.2
O3—In—O5	117.26 (6)	C17—C18—H18A	110.2
O1—In—O5	118.30 (6)	O5—C18—H18B	110.2
C2—In—O4	85.73 (10)	C17—C18—H18B	110.2
C1—In—O4	95.67 (9)	H18A—C18—H18B	108.5
O2—In—O4	120.85 (6)	O5—C19—C110	107.6 (2)
O3—In—O4	59.79 (6)	O5—C19—H19A	110.2
O1—In—O4	176.74 (6)	C110—C19—H19A	110.2
O5—In—O4	58.96 (6)	O5—C19—H19B	110.2
C2—In—O6	86.86 (11)	C110—C19—H19B	110.2
C1—In—O6	88.65 (9)	H19A—C19—H19B	108.5
O2—In—O6	121.12 (6)	O6—C110—C19	108.1 (3)
O3—In—O6	175.45 (6)	O6—C110—H11C	110.1
O1—In—O6	59.76 (6)	C19—C110—H11C	110.1
O5—In—O6	59.11 (6)	O6—C110—H11D	110.1
O4—In—O6	117.24 (6)	C19—C110—H11D	110.1
In—C1—H1A	109.5	H11C—C110—H11D	108.4
In—C1—H1B	109.5	O6—C111—C112	107.7 (3)
H1A—C1—H1B	109.5	O6—C111—H11E	110.2
In—C1—H1C	109.5	C112—C111—H11E	110.2
H1A—C1—H1C	109.5	O6—C111—H11F	110.2
H1B—C1—H1C	109.5	C112—C111—H11F	110.2
In—C2—H2A	109.5	H11E—C111—H11F	108.5
In—C2—H2B	109.5	O1—C112—C111	108.7 (3)
H2A—C2—H2B	109.5	O1—C112—H11G	109.9
In—C2—H2C	109.5	C111—C112—H11G	109.9
H2A—C2—H2C	109.5	O1—C112—H11H	109.9
H2B—C2—H2C	109.5	C111—C112—H11H	109.9
O1—C11—C12	107.9 (2)	H11G—C112—H11H	108.3
O1—C11—H11A	110.1	C112—O1—C11	112.3 (2)
C12—C11—H11A	110.1	C112—O1—In	117.95 (17)
O1—C11—H11B	110.1	C11—O1—In	112.53 (17)
C12—C11—H11B	110.1	C13—O2—C12	111.2 (2)
H11A—C11—H11B	108.4	C13—O2—In	114.95 (17)
O2—C12—C11	108.0 (2)	C12—O2—In	118.51 (17)
O2—C12—H12A	110.1	C14—O3—C15	111.3 (2)
C11—C12—H12A	110.1	C14—O3—In	116.02 (18)
O2—C12—H12B	110.1	C15—O3—In	120.42 (17)
C11—C12—H12B	110.1	C16—O4—C17	111.0 (2)
H12A—C12—H12B	108.4	C16—O4—In	114.27 (16)
O2—C13—C14	108.3 (3)	C17—O4—In	116.43 (16)
O2—C13—H13A	110.0	C19—O5—C18	111.2 (2)
C14—C13—H13A	110.0	C19—O5—In	118.53 (17)
O2—C13—H13B	110.0	C18—O5—In	116.76 (16)
C14—C13—H13B	110.0	C110—O6—C111	112.7 (2)
H13A—C13—H13B	108.4	C110—O6—In	112.93 (17)
O3—C14—C13	108.2 (3)	C111—O6—In	113.48 (17)
O3—C14—H14A	110.1	O22—S—O21	115.40 (16)
C13—C14—H14A	110.1	O23—S—O21	114.74 (15)
O3—C14—H14B	110.1	O22—S—O23	114.82 (15)
C13—C14—H14B	110.1	O21—S—C	103.03 (15)
H14A—C14—H14B	108.4	O22—S—C	103.32 (15)
O3—C15—C16	108.4 (2)	O23—S—C	103.08 (17)
O3—C15—H15A	110.0	F1—C—F2	108.2 (3)
C16—C15—H15A	110.0	F1—C—F3	107.3 (3)
O3—C15—H15B	110.0	F3—C—F2	107.4 (3)
C16—C15—H15B	110.0	F1—C—S	111.6 (3)
H15A—C15—H15B	108.4	F2—C—S	110.4 (3)
O4—C16—C15	107.7 (3)	F3—C—S	111.7 (2)
			
O1—C11—C12—O2	62.2 (3)	C18—C17—O4—C16	179.6 (2)
O2—C13—C14—O3	−61.1 (4)	C18—C17—O4—In	46.6 (3)
O3—C15—C16—O4	60.4 (3)	C2—In—O4—C16	118.6 (2)
O4—C17—C18—O5	−63.4 (3)	C1—In—O4—C16	−65.7 (2)
O5—C19—C110—O6	64.9 (3)	O2—In—O4—C16	32.9 (2)
O6—C111—C112—O1	−64.2 (3)	O3—In—O4—C16	18.94 (19)
C111—C112—O1—C11	−177.6 (3)	O1—In—O4—C16	−180 (100)
C111—C112—O1—In	49.0 (3)	O5—In—O4—C16	−146.8 (2)
C12—C11—O1—C112	172.2 (3)	O6—In—O4—C16	−157.14 (19)
C12—C11—O1—In	−51.9 (3)	C2—In—O4—C17	−109.9 (2)
C2—In—O1—C112	68.0 (2)	C1—In—O4—C17	65.8 (2)
C1—In—O1—C112	−107.7 (2)	O2—In—O4—C17	164.43 (18)
O2—In—O1—C112	154.9 (2)	O3—In—O4—C17	150.4 (2)
O3—In—O1—C112	167.2 (2)	O1—In—O4—C17	−48.3 (11)
O5—In—O1—C112	−25.4 (2)	O5—In—O4—C17	−15.28 (18)
O4—In—O1—C112	6.6 (11)	O6—In—O4—C17	−25.6 (2)
O6—In—O1—C112	−16.8 (2)	C110—C19—O5—C18	176.8 (2)
C2—In—O1—C11	−65.2 (2)	C110—C19—O5—In	−43.6 (3)
C1—In—O1—C11	119.0 (2)	C17—C18—O5—C19	−168.3 (2)
O2—In—O1—C11	21.60 (18)	C17—C18—O5—In	51.4 (3)
O3—In—O1—C11	34.0 (2)	C2—In—O5—C19	−73.2 (2)
O5—In—O1—C11	−158.67 (17)	C1—In—O5—C19	103.3 (2)
O4—In—O1—C11	−126.7 (10)	O2—In—O5—C19	151 (11)
O6—In—O1—C11	−150.0 (2)	O3—In—O5—C19	−171.05 (18)
C14—C13—O2—C12	−170.3 (3)	O1—In—O5—C19	20.8 (2)
C14—C13—O2—In	51.6 (3)	O4—In—O5—C19	−157.2 (2)
C11—C12—O2—C13	−179.8 (3)	O6—In—O5—C19	12.09 (17)
C11—C12—O2—In	−43.3 (3)	C2—In—O5—C18	64.1 (2)
C2—In—O2—C13	−118.9 (2)	C1—In—O5—C18	−119.4 (2)
C1—In—O2—C13	64.5 (2)	O2—In—O5—C18	−71 (11)
O3—In—O2—C13	−21.2 (2)	O3—In—O5—C18	−33.72 (19)
O1—In—O2—C13	147.2 (2)	O1—In—O5—C18	158.14 (18)
O5—In—O2—C13	16 (11)	O4—In—O5—C18	−19.85 (18)
O4—In—O2—C13	−34.8 (2)	O6—In—O5—C18	149.4 (2)
O6—In—O2—C13	155.66 (19)	C19—C110—O6—C111	174.8 (2)
C2—In—O2—C12	106.2 (2)	C19—C110—O6—In	−55.0 (3)
C1—In—O2—C12	−70.4 (2)	C112—C111—O6—C110	178.9 (2)
O3—In—O2—C12	−156.1 (2)	C112—C111—O6—In	48.9 (3)
O1—In—O2—C12	12.29 (19)	C2—In—O6—C110	117.1 (2)
O5—In—O2—C12	−119 (11)	C1—In—O6—C110	−62.1 (2)
O4—In—O2—C12	−169.71 (18)	O2—In—O6—C110	−156.56 (17)
O6—In—O2—C12	20.8 (2)	O3—In—O6—C110	−14.6 (8)
C13—C14—O3—C15	−174.9 (3)	O1—In—O6—C110	−147.9 (2)
C13—C14—O3—In	42.4 (3)	O5—In—O6—C110	23.21 (17)
C16—C15—O3—C14	173.5 (3)	O4—In—O6—C110	33.55 (19)
C16—C15—O3—In	−45.7 (3)	C2—In—O6—C111	−113.0 (2)
C2—In—O3—C14	72.7 (2)	C1—In—O6—C111	67.7 (2)
C1—In—O3—C14	−108.3 (2)	O2—In—O6—C111	−26.7 (2)
O2—In—O3—C14	−12.3 (2)	O3—In—O6—C111	115.2 (7)
O1—In—O3—C14	−24.6 (2)	O1—In—O6—C111	−18.03 (19)
O5—In—O3—C14	167.9 (2)	O5—In—O6—C111	153.1 (2)
O4—In—O3—C14	154.1 (2)	O4—In—O6—C111	163.42 (19)
O6—In—O3—C14	−155.8 (7)	O22—S—C—F1	58.2 (3)
C2—In—O3—C15	−66.4 (2)	O23—S—C—F1	−61.6 (3)
C1—In—O3—C15	112.6 (2)	O21—S—C—F1	178.7 (3)
O2—In—O3—C15	−151.5 (2)	O22—S—C—F3	−61.9 (3)
O1—In—O3—C15	−163.7 (2)	O23—S—C—F3	178.2 (2)
O5—In—O3—C15	28.8 (2)	O21—S—C—F3	58.6 (3)
O4—In—O3—C15	15.0 (2)	O22—S—C—F2	178.6 (3)
O6—In—O3—C15	65.1 (8)	O23—S—C—F2	58.7 (3)
C15—C16—O4—C17	177.6 (2)	O21—S—C—F2	−60.9 (3)
C15—C16—O4—In	−48.3 (3)		

### Hydrogen-bond geometry (Å, °)

**Table:**

*D*—H···*A*	*D*—H	H···*A*	*D*···*A*	*D*—H···*A*
C13—H13B···O22^i^	0.99	2.54	3.455 (4)	153
C14—H14A···O23^ii^	0.99	2.43	3.348 (4)	155
C17—H17A···O5^iii^	0.99	2.58	3.527 (4)	160
C19—H19A···O23^iv^	0.99	2.53	3.322 (4)	137

Symmetry codes: (i) −*x*+1, −*y*+1, −*z*; (ii) −*x*+1, *y*−1/2, −*z*+1/2; (iii) −*x*+2, −*y*, −*z*+1; (iv) −*x*+2, −*y*+1, −*z*+1.
